# Dengue Virus RNA Structure Specialization Facilitates Host Adaptation

**DOI:** 10.1371/journal.ppat.1004604

**Published:** 2015-01-30

**Authors:** Sergio M. Villordo, Claudia V. Filomatori, Irma Sánchez-Vargas, Carol D. Blair, Andrea V. Gamarnik

**Affiliations:** 1 Fundación Instituto Leloir-CONICET, Buenos Aires, Argentina; 2 Arthropod-borne and Infectious Diseases Laboratory, Department of Microbiology, Immunology and Pathology, Colorado State University, Fort Collins, Colorado, United States of America; University of Kentucky, UNITED STATES

## Abstract

Many viral pathogens cycle between humans and insects. These viruses must have evolved strategies for rapid adaptation to different host environments. However, the mechanistic basis for the adaptation process remains poorly understood. To study the mosquito-human adaptation cycle, we examined changes in RNA structures of the dengue virus genome during host adaptation. Deep sequencing and RNA structure analysis, together with fitness evaluation, revealed a process of host specialization of RNA elements of the viral 3’UTR. Adaptation to mosquito or mammalian cells involved selection of different viral populations harvesting mutations in a single stem-loop structure. The host specialization of the identified RNA structure resulted in a significant viral fitness cost in the non-specialized host, posing a constraint during host switching. Sequence conservation analysis indicated that the identified host adaptable stem loop structure is duplicated in dengue and other mosquito-borne viruses. Interestingly, functional studies using recombinant viruses with single or double stem loops revealed that duplication of the RNA structure allows the virus to accommodate mutations beneficial in one host and deleterious in the other. Our findings reveal new concepts in adaptation of RNA viruses, in which host specialization of RNA structures results in high fitness in the adapted host, while RNA duplication confers robustness during host switching.

## Author Summary

Important viral pathogens, such as influenza and dengue, jump between species; however, it is still unclear how these viruses evolved for efficient replication in significantly different environments. Using dengue virus as a model, which naturally alternates between humans and mosquitoes, changes in the viral RNA were investigated in each host. Deep sequencing analysis revealed the selection of strikingly different viral populations during host adaptation. Fitness measurements indicated that mutations in a single RNA structure of the viral 3’ untranslated region were responsible for positive and negative selection of specific viral variants in the two hosts. Cycles of disruption and reconstitution of this RNA structure were observed during host switching, identifying a host adaptable RNA element. Importantly, natural duplication of this RNA was found to be required to tolerate mosquito-associated mutations for efficient replication in mammalian cells. Our studies revealed a novel strategy of viral adaptation, where RNA structure specialization and duplication provide a mechanism for maintaining high viral fitness in each host and efficiency during host cycling. Because the identified RNA structure and its duplication are conserved in many mosquito-borne flaviviruses, our findings using dengue virus could help to understand RNA evolution of an extensive group of human pathogens.

## Introduction

Arboviruses infect vertebrate and invertebrate hosts. This natural process of crossing between species raises a number of questions about the ability of these viruses to use different cellular machineries and overcome different types of antiviral responses. RNA viruses in general have high capacity to adapt to different environments due to the genetic diversity of viral populations [1, 2]. One of the consequences of this ability for adaptation is the emergence of new pathogenic viruses [3]. It has been assumed that viruses that naturally alternate between different hosts evolve less rapidly than those that specialize in a single host [4]. This evolutionary constraint can be attributed primarily to the obligate cycling between hosts with conflicting demands for viral replication, where sequence changes that improve fitness in one host may not be advantageous or might even be deleterious in the other host [5]. Several studies using arboviruses support the idea that releasing a virus from host alternation could result in host specialization with a fitness cost in the bypassed host (for reviews see [6, 7]). Although a great deal of information on this subject has been accumulated using both in vivo and in vitro systems, understanding the molecular aspects of host specialization and the genetic determinants of the fitness cost associated with host alternation remain a major challenge. Using dengue virus (DENV), a member of the *Flaviviridae* family that cycles between mosquitoes and humans, we recently found specific RNA sequences in the viral 3’UTR that are essential for viral replication in mosquito cells but dispensable for replication in mammalian cells [8]. These studies provided direct evidence for host-specific functions of viral RNA elements and raised the question whether viral RNA structures are under specific selective pressures during host adaptation.

The DENV genome is a dynamic RNA molecule that adopts linear and circular conformations in the infected cell. This RNA contains a great deal of information in cis-acting RNA structures that enhance, suppress and/or promote viral replication [9]. The viral 5’UTR includes two essential elements for DENV replication in mosquito and mammalian cells, the promoter for RNA synthesis known as stem-loop A, and a genome cyclization sequence [10]. The general organization of the DENV 3’UTR is similar to that of other flaviviruses, containing essential elements for viral replication and accessory RNA structures that modulate viral processes. An interesting feature of the 3’UTR of all flaviviruses is the presence of short direct repeats (DRs) and long RNA element duplications [11]. The function of these duplications is still unclear, but previous evolutionary studies have suggested direct association of these RNA elements with viral adaptation to multiple invertebrate and vertebrate hosts [12].

DENV is geographically expanding and increasing in virulence, while neither vaccines nor specific antivirals are available. Accordingly, understanding the determinants and limitations of alternating between humans and mosquitoes will be important for developing effective antiviral measures. Here, we describe how DENV RNA cis-acting elements evolve under single-host selective pressures in mosquito and mammalian cells. Deep sequencing of DENV populations subjected to host adaptation showed a strong selection of specific mutations in the viral 3’UTR in each host, while cis-acting elements present at the 5’ end of the genome remained unchanged. Secondary RNA structure analysis showed that viruses selected in mosquito cells contained mutations mapping to a single stem-loop (SL) structure. Fitness of recombinant viruses carrying the identified mutations showed conflicting requirements for replication in human and mosquito cells. Cycles of disruptions and reconstitutions of the identified SL structure were observed during host switching, defining a host-adaptable RNA structure. These RNA changes observed during experimental adaptation recapitulated the selection of mutations observed in natural DENV isolates from humans and mosquitoes. Phylogenetic analysis of structural features of 3’UTRs of mosquito-borne flaviviruses (MBVF) revealed a conserved duplication of the host-adaptable SL structure, which is absent in insect-specific flaviviruses. Interestingly, using viruses with single or duplicated SLs, we found that RNA duplication is required to tolerate mosquito-associated mutations for replication in mammalian cells, where these mutations are deleterious. We suggest a novel model for host-specific viral adaptation where RNA specialization and duplication provide a mechanism for maintaining high viral fitness in each host and robustness during vector-host cycling.

## Results

### Dynamics of DENV populations during host cell adaptation

To explore host-specific adaptation of RNA structures in the DENV genome, we examined nucleotide sequence variations in DENV populations restricted to replicate in mosquito or mammalian cells for 20 consecutive passages. Viral populations were obtained by transfection of an in vitro transcribed RNA from the infectious DENV2 cDNA clone pD2-IC 30-PA [13], originally isolated from a patient with dengue hemorrhagic fever (*Asian* genotype). The first viral populations obtained in mosquito C6/36 and mammalian BHK cells were named passage 1 (P1). The P1 virus from each cell type was then serially passaged to generate P5, P10, P15 and P20 in the same cell line. Two independent adaptation experiments were performed simultaneously. Amplicons corresponding to the genomic 5’ end and 3’UTR of each population were obtained and used for deep sequencing. Raw sequencing data were processed following a work-flow described in Experimental Procedures. Viral variants in the population were defined using statistical and clustering approaches. A cluster of sequences was considered a different variant when its frequency in the population was 1% or higher.

**Figure 1:**
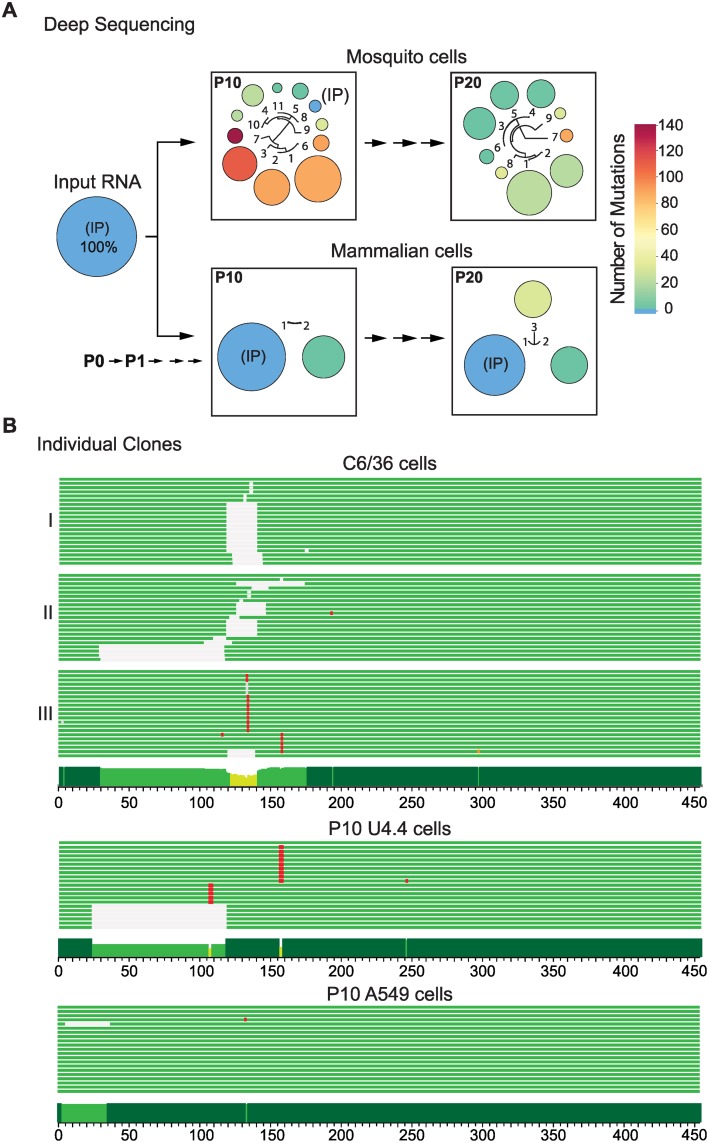
Sequence variations at the DENV 3’UTR during experimental host adaptation. **(A)** Deep sequencing of viral populations after 10 and 20 passages (P10 and P20) in mosquito and mammalian cells. The area and color of circles represent the frequency and number of mutations of the viral variants, respectively. A schematic fan dendrogram indicating the distance between the variants for each population is also shown. For nucleotide sequences and frequencies of each viral variant also see S1 Fig. **(B)** Sequence of cloned amplicons corresponding to the complete 3’UTR. For each P10 population obtained, 20 individual clones were sequenced. Cells lines used for adaptation experiments are indicated (mosquito C6/36, mosquito U4.4, and human A549 cells). Mutations are indicated in red and deletions in grey. A conservation plot is presented at the bottom. The three independent experiments in C6/36 cells are indicated on the left (I, II and III).

The 3’UTR of the in vitro transcribed input RNA was sequenced as reference (IP). After 10 passages in mosquito cells (P10), the IP 3’UTR sequence changed from a frequency of 100% to 2.3% (Fig. 1A). Viral variants are represented by circles; the size and color of the circles indicate the frequency and number of nucleotide changes, respectively. Interestingly, 10 different variants that contained point mutations and deletions were significantly enriched in mosquito cells. Viral genomes with deletions corresponding to the 5’ proximal region of the 3’UTR were found with frequencies of 34, 21 and 18% (P10, variants 1, 2 and 3, respectively, Fig. 1A). Nucleotide sequences, frequencies and descriptions of variants found are shown in S1 Fig. After 20 passages in mosquito cells (P20), 9 different variants were enriched in the population and the IP sequence was undetectable (Fig. 1A). Moreover, the large deletions observed in P10 were replaced by shorter deletions and point mutations mainly restricted to nucleotides 117 to 156 of the 3’UTR (S1A Fig.) (position 1 refers to the first nucleotide after the translation stop codon). In contrast, when the virus was successively passaged in mammalian cells (P10 and P20), only few viral variants were selected. For example, two clusters were observed for P10: the IP sequence (72%) and a variant with a mutation at position 59 (28%) (Fig. 1A and S1A Fig.). The sequencing data of an independent adaptation experiment is shown in S2A and S2B Figs.

Viral 5’UTRs from adapted populations were also analyzed. Amplicons corresponding to the first 200 nucleotides of the DENV genome, including the 5’UTR and *cis*-acting RNA elements in the capsid coding region were sequenced. No mutations were enriched in any population obtained after adapting the virus to mammalian or mosquito cells, indicating an important constraint for evolution in this region of the genome (S2C Fig.). The data highlight a fundamentally different response of the viral 5’ and 3’UTRs to host adaptation. While a large number of mutations were quickly accumulated in the 3’UTR, 5’ end sequences remained stable.

To confirm results from the deep sequencing data that indicated profound changes in the viral 3’UTR, individual clones obtained from independent adaptation experiments were cloned and sequenced conventionally. The two P10 populations and a P20 obtained in mosquito C6/36 cells were used for this analysis. Twenty clones of the 3’UTR from each sample were sequenced. Mutations were found in all sixty clones while the IP sequence was undetectable (Fig. 1B). Experiment II in Fig. 1B corresponds to the P10 population shown in Fig. 1A. Many of the mutations observed by conventional and deep sequencing were the same (S3A Fig.). Interestingly, the evolutionary pathway for viral variant selection was different in independent adaptation experiments, but in all the cases mutations clustered within the same region (Fig. 1B, C6/36 and S3A Fig.). Analysis of cloned 3’UTRs was also performed in independent adaptation experiments in mammalian BHK cells. In this case, the input sequence was maintained with high frequency and a mutation at position 59 was observed (S3B Fig.). DENV adaptation was also examined in human A549 and mosquito U4.4 cells, which are fully functional for IFN and RNAi responses, respectively [14]. At P10 in mosquito U4.4 cells, the input sequence was undetectable and, as observed in C6/36 cells, point mutations and deletions were selected (Fig. 1B). In contrast, no notable increase in frequency of viral variants with mutations in the 3’UTR was observed in human A549 cells.

These results indicate that the composition of DENV populations in mosquito and mammalian cells are strikingly different and that enrichment of variants containing mutations in the 3’UTR is host-specific.

### DENV 3’UTR mutations are responsible for large replication differences in the two hosts

DENV replication in mosquito cells resulted in the selection of viral variants with mutations in the viral 3’UTR. To examine the replication advantage of selected variants, a cloned 3’UTR from a virus obtained at high frequency in the adaptation experiments (variant 1 in P10 of Fig. 1A and S1A Fig.) was introduced into the parental virus, and replication parameters in both host cell types were evaluated.

**Figure 2:**
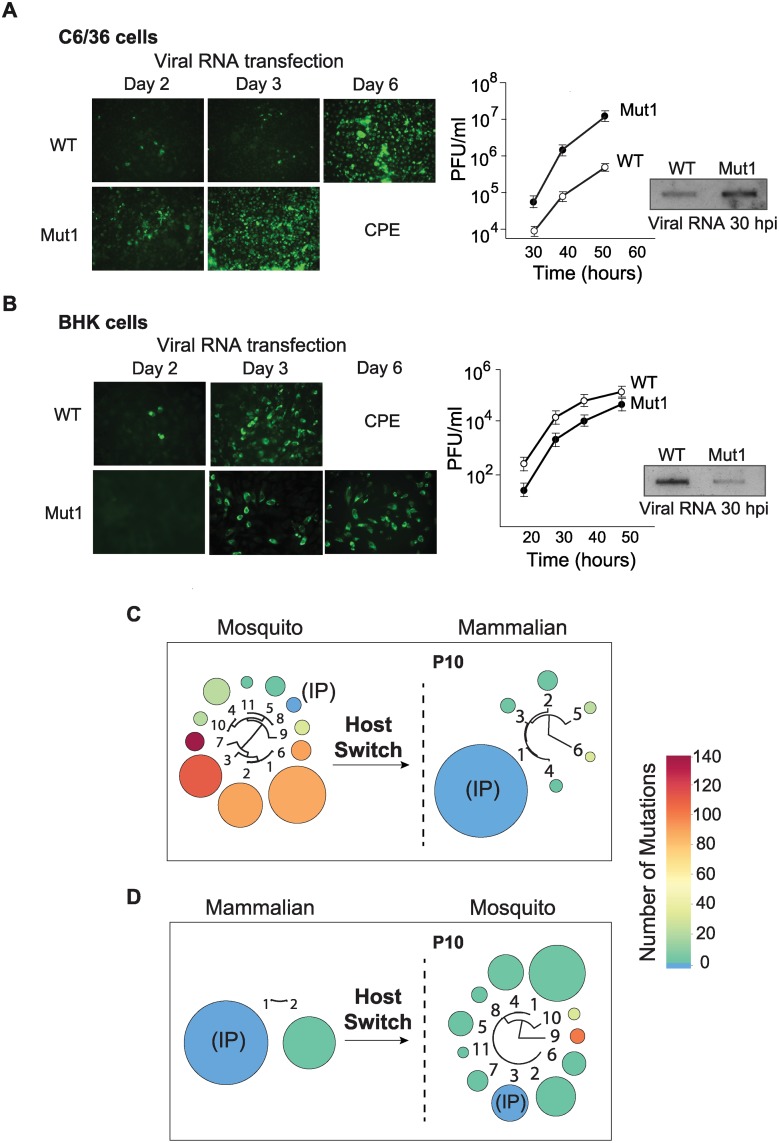
Fitness parameters and evolution of viral populations after host switch indicate that the DENV 3’UTR is under host-specific selective pressure. **(A)** Immunofluorescence and growth kinetics of recombinant virus carrying the 3’UTR of a variant selected in mosquito cells (Mut1) compared with the parental virus (WT) performed in C6/36 cells. Cytopathic effect (CPE) is indicated. Inset shows the accumulation of specific viral RNA. **(B)** Fitness studies in BHK cells for the two viruses shown in A. **(C)** and **(D)** Deep sequencing of viral populations passaged 10 successive times in mammalian or mosquito cells obtained after host switch as indicated. For nucleotide sequences and frequencies of each viral variant also see S4 Fig.

RNA transcripts from WT virus or that containing the 3’UTR of variant 1 (Mut1) were individually transfected into BHK or C6/36 cells, and immunofluorescence (IF) was followed as a function of time. Replication of Mut1 showed a clear advantage in mosquito cells compared to WT. The cell monolayer was completely infected with Mut1 at day 3, while the WT only infected 10 to 20% of the cells at the same time point (Fig. 2A). Mut1 also exhibited enhanced growth kinetics, resulting in 10 to 100 fold higher titers as compared to WT (Fig. 2A). Viral genome accumulation in one replication cycle was also measured by specific radiolabeled probes, showing significantly higher levels of viral RNA for the Mut1 virus (Fig. 2A). Similar analyses of Mut1 and WT viruses were carried out in mammalian cells. Interestingly, Mut1 appeared to have a disadvantage for replication in BHK cells (Fig. 2B).

The results indicate that the observed mutation in the 3’UTR was beneficial for viral replication in mosquito cells but seemed to be detrimental in mammalian cells. If this were the case, positive and negative selection of different viral variants could take place during host switch and the frequencies of mutants in the population would change. To examine this possibility and explore how the host cell environment forces changes in the composition of the population, we used deep sequencing after host switches. The P10 population derived from the mosquito cell adaptation experiment was subjected to ten passages in mammalian cells. The resulting progeny showed a dramatic change in composition, in which almost all the variants collapsed into the IP sequence (Fig. 2C). While the initial mosquito-adapted population contained only 2.3% of the IP sequence, after host switch this IP sequence became 89% of the population (Fig. 2C). In addition, variants with large deletions that occurred at high frequency in the mosquito population decreased. For instance variants 1, 2 and 3 with deletions in the 3’UTR that made up 73% of the mosquito-adapted population became undetectable (below 1%) after the host switch. Interestingly, when P10 from mammalian cells was switched to mosquito cells for ten passages, a diversification of the population was observed. Eleven different viral variants with point mutations and short deletions emerged. In this case, variants with 1, 2, and 3 nucleotide substitutions were selected (Fig. 2D); also see S4 Fig. for nucleotide sequence and frequency of each selected variant.

These experiments showed that the DENV population is dynamic after host switch and that different variants are positively or negatively selected. In addition, the replication of recombinant DENVs carrying cloned selected 3’UTR sequences confirmed that this region of the viral genome was responsible for large viral fitness differences in the two hosts.

### Two conserved stem-loop structures in the variable region of the DENV 3’UTR

**Figure 3:**
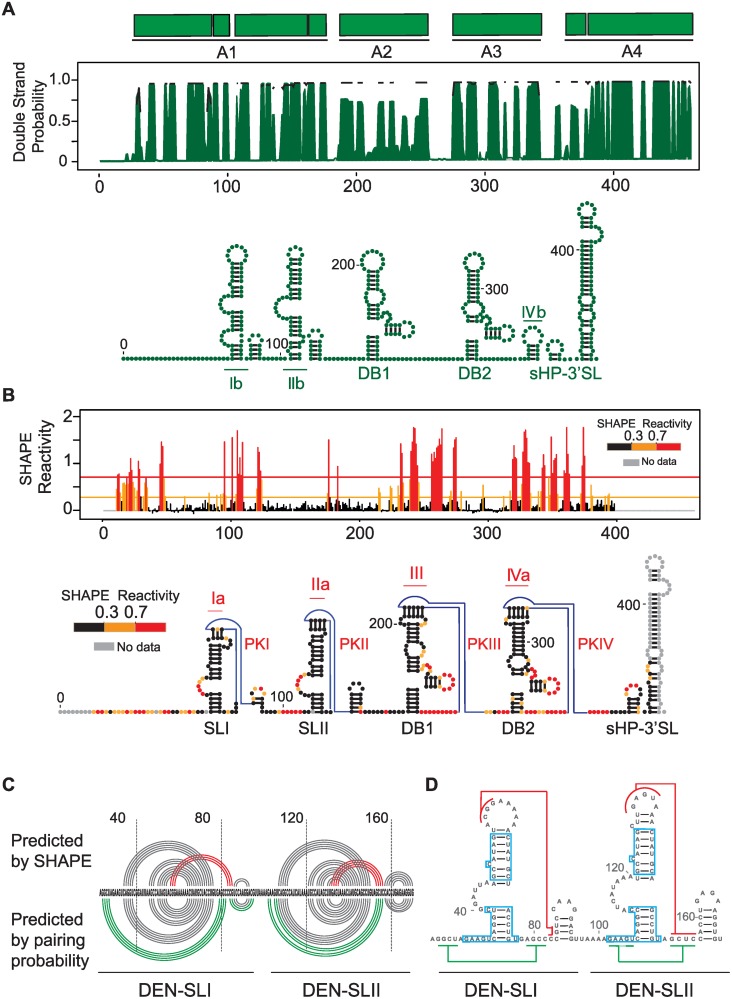
Structural organization of the 3’UTR of dengue virus. **(A)** Secondary structure model predicted by conservation and stability. Different DENV type 2 genotypes were analyzed using RNAalifold and RNAz softwares. A plot with the base pairing probability calculated for the A1, A2, A3 and A4 domains (top) and the most stable conserved RNA structures (bottom) are shown. **(B)** Secondary and tertiary structures assessed by chemical probing. SHAPE reactivity plot (top) and predicted RNA structure model for DENV 3’UTR (bottom) are shown. An unstructured non-conserved region of 23 nucleotides is followed by two SLs (SLI and SLII) and two dumbbell (DB1 and DB2) structures. Four pseudoknots are predicted as indicated (PKI, PKII, PKIII and PKIV). **(C)** Comparison between structure conservation and SHAPE prediction of DEN-SLI and DEN-SLII. Predicted pseudoknots and additional hybridization at the base of the stem-loops are shown with red and green lines, respectively; indicating the existence of two alternative conformations. **(D)** Regions with identical sequences in DEN-SLI and DEN-SLII are shown (blue boxes), suggesting a common origin of these two RNA structures.

Because all the mutations selected during DENV host adaptation experiments were located in the variable region of the viral 3’UTR, we examined the conservation and the secondary structure of this part of the genome in greater detail. First, we used an evolutionary conservation analysis that takes into account the rate and pattern of nucleotide changes in DENV sequence alignments and predicts the likelihood that a particular nucleotide is involved in a base-pair interaction (double strand probability, Fig. 3A). As expected, high levels of conservation within the short hairpin 3’ stem-loop (sHP-3’SL) and the dumbbell structures (DBs), also known as A4 and A2-A3 regions, respectively, were observed. Strong conservation was also detected in the variable region, also known as A1. Thermodynamic and evolutionary folding predictions generated two conserved large stem-loop structures and two short hairpins in the A1 region (Fig. 3A).

Next, we probed the 3’UTR in the context of the whole viral genome with a SHAPE reagent, *N*-methylisotoic anhydride (NMIA). The reactivity plot of the 3’UTR shows accurate formation of the two DB structures with a clear correlation with previous reports of paired and unpaired nucleotides [15, 16], including the formation of two pseudoknots (PKIII and PKIV) (Fig. 3B). Within the A1 region, the SHAPE reactivity supports the presence of two stem loops, here named DEN-SLI and DEN-SLII. It is important to clarify that these two SLs correspond to the previously described SLII and SLIV for WNV, respectively [17] (see discussion for further explanation of the proposed acronyms). Low reactivity of top-loop nucleotides and the presence of unreactive complementarity in downstream sequences predict the formation of two additional PKs (PKI and PKII, Fig. 3B), in agreement with data recently reported using individual RNA domains [18].

A significant correlation was observed when the pairing probability was combined with the SHAPE reactivity (Fig. 3C). However, the base of DEN-SLI and DEN-SLII structures were predicted to form longer stems (four extra base pairs) by conservation analysis (Ib and IIb, Fig. 3A), while SHAPE studies supported formation of PKI and PKII (Ia and IIa, Fig. 3B). Based on these studies, it is likely that both mutually-exclusive structures within DEN-SLI and DEN-SLII can form independently (Fig. 3C). Interestingly, sequence comparison between the two SLs indicated the presence of regions with identical nucleotide sequences (boxed nucleotides, Fig. 3D). This observation suggests a duplication as origin of these RNA elements.

### Host specialization of DENV RNA elements in cell culture recapitulates selection of natural variants isolated from humans and mosquitoes

**Figure 4:**
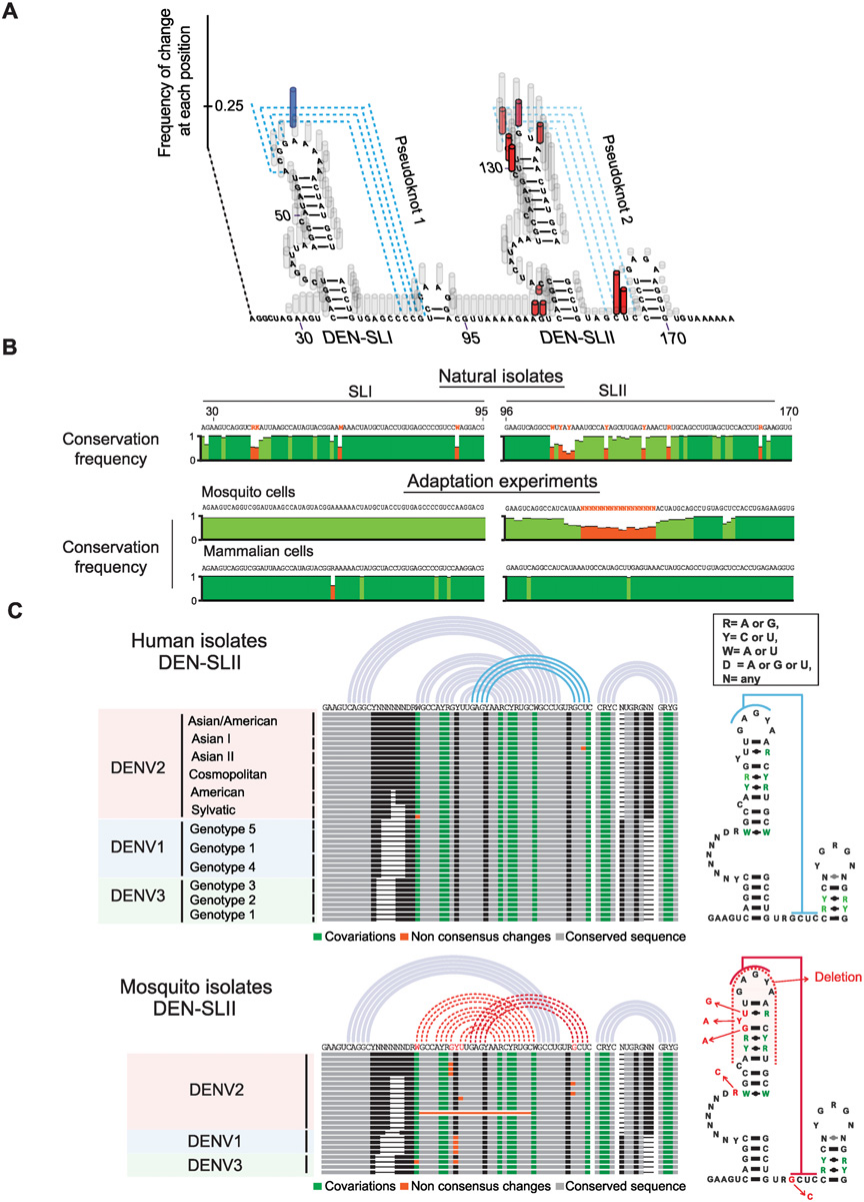
Nucleotide variations detected in experimentally adapted viruses correlate with mutations found in natural isolates. **(A)** Adaptive mutations and deletions were mapped on the RNA structure of the variable region of the viral 3’UTR. Deletions and point mutations rescued from mosquito cell-adapted populations are indicated in grey and red, respectively. Mutation selected in mammalian cells is shown in blue. **(B)** Conservation plots comparing variable regions of cell adapted viruses and natural isolates. **(C)** Representation of SL-II RNA structures of DENV genomes from natural isolates corresponding to different serotypes. Sequence alignment plots and secondary RNA structure models are shown for DENV isolates from humans (top) and mosquitoes (bottom).

To understand the significance of variant selection during viral adaptation in mosquito and mammalian cells, we mapped the nucleotide changes found in the adaptation experiments into the predicted RNA model. A plot of frequency of changes for each position of the viral RNA was generated (Fig. 4A). In mammalian cells, the only mutation consistently selected was A59G, which mapped to the top loop of DEN-SLI and was predicted to stabilize PKI by an additional GC base pair. In mosquito cells, genomes with large deletions, including sequences within DEN-SLI and DEN-SLII structures, were observed. However, high frequency variants contained point mutations and deletions that mapped exclusively to DEN-SLII, between nucleotides A104 and U155, disrupting either the structure of DEN-SLII or its ability to form PKII (Fig. 4A). This observation was particularly interesting because both SLs have similar sequences and structures; however, they are under remarkably different selective pressures in the two hosts.

Sequence alignments of DEN-SLI and DEN-SLII from genomes obtained in the adaptation experiments were compared with the respective sequences from natural DENV2 isolates. The sequence and structure of DENV-SLI was conserved, although with variability in the side-loop, which is under neutral selection. In contrast, DEN-SLII showed extensive sequence variability between nucleotides 112 and 142 (Fig. 4B). Interestingly, this variability observed in natural isolates maintained the structure of SLII by co-variations (see below). To identify the source of DEN-SLII variability, DENV isolates were separated according to the host samples from which they were originally obtained, either human or mosquito. Sequence alignments of DENV genotypes of different serotypes were used. DENV4 was not included in this analysis because it has a single SL with unique structural properties. This RNA structure has some features of SLI and some of SLII and was analyzed separately (S5A Fig.). Sequence alignments of DEN-SLII of DENV1, DENV2 and DENV3 obtain from humans support high conservation of this structure, in which all the sequence variations are consistent with co-variations that maintained the stem-loop (Fig. 4C). In contrast, sequence alignments of DENVs isolated from mosquitoes reveal mutations in DEN-SLII that change conserved nucleotides or disrupt the structure (Fig. 4C), strikingly similar to those observed in experimental adaptation to mosquito cells (Fig. 4A). The sequence alignment of mosquito isolates included viruses obtained from different *Aedes* spp. mosquitoes (*A. aegypti, A. albopictus, A. luteocephalus, A. africanus* and *A. niveus*). A list of sources of sequences used for this analysis is included in S1 Table. The results indicate that experimental adaptation in cell culture selects viral variants with mutations or reconstitutions of DEN-SLII, which recapitulates that observed in natural isolates from mosquitoes and humans.

### Functional relevance of DEN-SLI and SLII in mosquito and human cells

**Figure 5:**
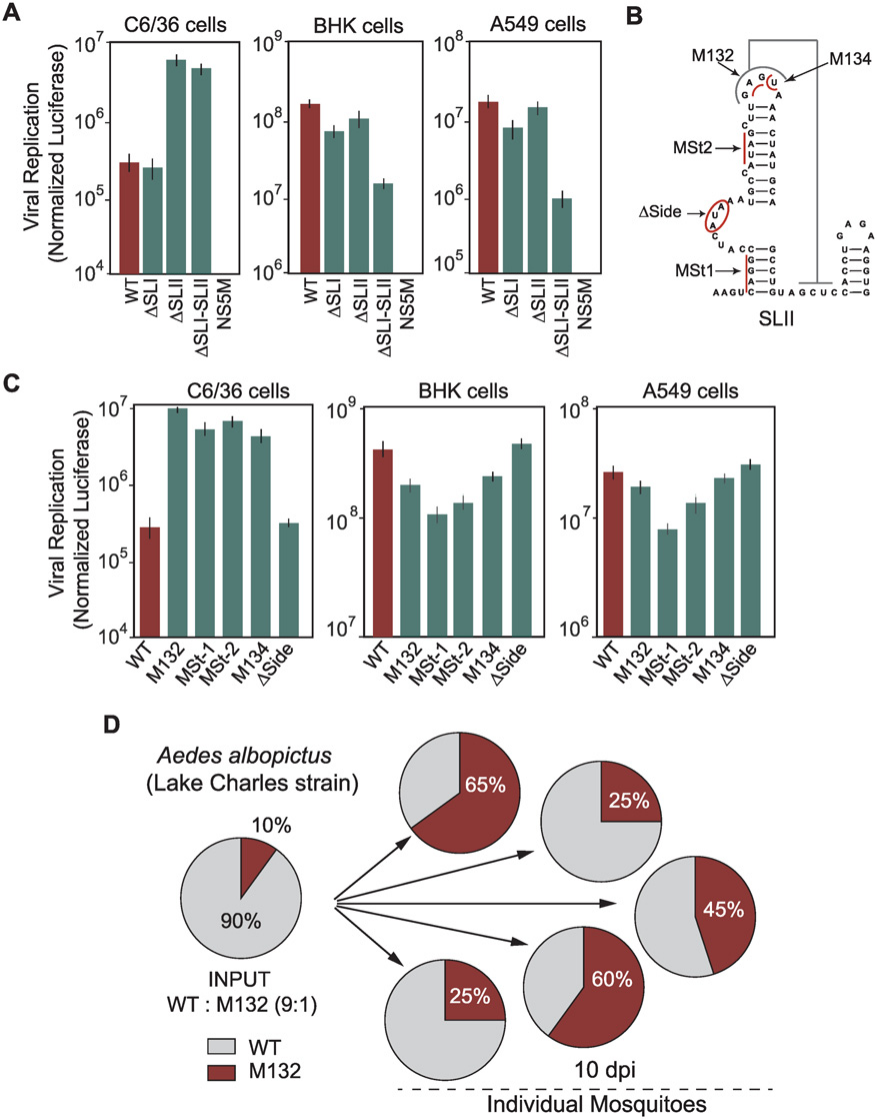
Requirements of DEN-SLI and DEN-SLII for viral replication in mosquito and human cells. **(A)** RNAs of reporter DENVs with deletions ΔSLI, ΔSLII or ΔSLI-II were transfected along with WT and replication impaired NS5M controls into C6/36, BHK and human A549 cells. Normalized luciferase levels are shown using a logarithmic scale at 48 h post transfection. The luciferase values are the mean +/- SD, n = 4. **(B)** Schematic representation of mutations in the DEN-SLII introduced in the reporter DENV. Red lines indicate substitutions and the circle indicates a deletion. **(C)** Viral RNA replication as in (A) for DEN-SLII mutants. The luciferase values are the mean +/- SD, n = 4. **(D)** Competition experiments in *A. albopictus* mosquitoes indicate a fitness advantage of variants with DEN-SLII mutations. Mosquitoes were injected with a mixture of the two viruses (WT: M132) at a 9:1 ratio and on day 10 individual mosquitoes were collected for viral RNA extraction and sequencing analysis of viral 3’UTRs. Pie charts with frequencies of viruses obtained from each mosquito are shown.

To investigate the function of DEN-SLI and DEN-SLII, the requirement of these RNA structures in viral replication was evaluated. Viruses carrying a deletion of each or both structures were constructed in a DENV reporter system [19]. RNA of ΔSLI, ΔSLII or ΔSLI-II mutants were transfected into C6/36, BHK, and human A549 cells, in addition to WT and a replication impaired (NS5M) controls. Viral RNA amplification was evaluated by luciferase activity 48 h post transfection. Interestingly, in mosquito C6/36 cells, ΔSLII and ΔSLI-II viruses amplified the viral RNA 23 and 20 fold more efficiently than WT, respectively (Fig. 5A), while ΔSLI RNA amplification levels were similar to WT. These results explain the selection of mutations exclusively in the SLII structure in the adaptation experiments. Replication of the mutants in BHK and human cells showed a remarkably different requirement. Deletion of both DEN-SLI-II greatly reduced viral replication (Fig. 5A). However, deletion of DEN-SLI decreased about 3-fold viral replication and deletion of DEN-SLII resulted in only a slight reduction of RNA replication. These results indicate that the two SLs have different functions in mosquito cells, while in human cells they appear to be functionally redundant. However, conserving both SLs appears to confer some benefit for replication in human cells.

To further characterize host-specific requirements of DEN-SLII, mutations in this structure observed in experimental adaptations and in viral isolates from mosquitoes were introduced in the reporter DENV. Viral genomes with mutation at the top loop (M132), a deletion of side loop nucleotides (Δside), substitutions disrupting bottom and top stems (MSt1 and MSt2, respectively), and a substitution disrupting PKII formation (M134) were designed (Fig. 5B). The results indicate that mutations within DEN-SLII resulted in a large increase in viral RNA replication in C6/36 mosquito cells (Fig. 5C). Mutations M132, MSt-1, MSt-2, and M134 increased viral RNA amplification between 15- and 36-fold in mosquito cells, while they reduced replication in BHK and human cells (Fig. 5C). Because DENV4 has only one SL with unique properties, we examined the function of this SL in DENV2 chimeras carrying the variable region of the 3’UTR of DENV4. This virus was able to replicate efficiently in both host cells, however, when subtle mutations were introduced debilitating the PK, viral replication in mammalian cells dropped about 30 fold (S5 Fig.), suggesting that DENV4 with a single SL would have fewer possibilities to accommodate mutations without an important fitness reduction.

Next, we examined the fitness of viruses with mutations selected in mosquito or human cells in adult mosquitoes. A recombinant virus was generated with the 3’UTR M132 mutation in the context of the DENV2 cDNA clone and used for competition experiments with a virus that efficiently replicated in human cells (intact SLII, WT virus). *Aedes albopictus* mosquitoes were intrathoracically inoculated with a mixture of WT and M132, 9:1, based on infectious viral titers. The frequency of the two viral variants in individual mosquitoes was evaluated at 10 days post-injection by sequencing 100 clones of the 3’UTR. This analysis showed a significant increase in frequency of mutant M132 over the WT in all mosquitoes (Fig. 5D). The results indicate an evident viral fitness advantage of a virus with a mutated SLII for replication in mosquitoes.

We conclude that host-adaptive mutations differentially modulate viral replication in the two hosts. In addition, while the two SLs play different functions in mosquito cells, the intact SLI and SLII appear to be redundant for viral replication in human cells.

### SL duplication in the viral 3’UTR reduces the fitness cost during host change

**Figure 6:**
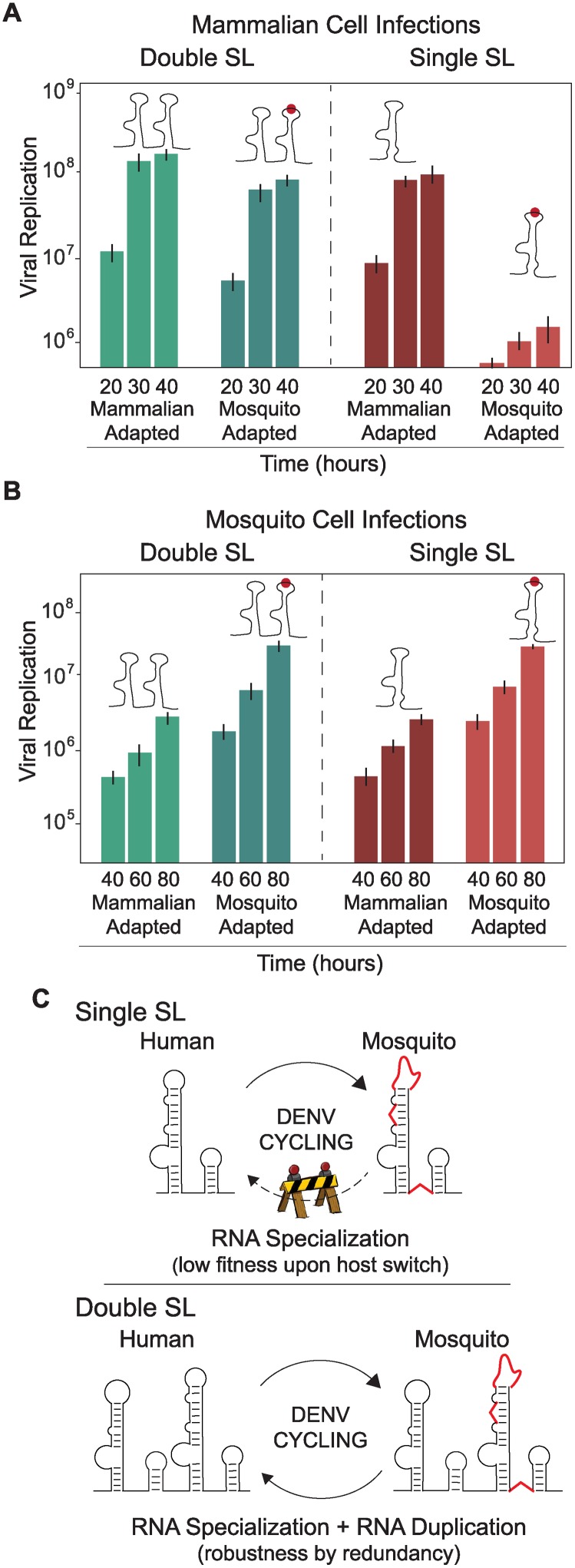
Fitness advantage of RNA structure duplication during DENV host switching. **(A)** Normalized luciferase levels expressed by reporter DENV with or without mutations resembling adaptations in mosquito or mammalian cells, respectively, carrying one or two SLs are shown for mammalian cells. The luciferase values are the mean +/- SD, n = 4. **(B)** The same as in **(A)** using mosquito cells. **(C)** Schematic representation of adaptable RNA structures emulating host switching of viruses with single or double SLs. Viruses with a single SL encounter a fitness barrier in the transition from mosquito to mammalian cells due to accumulation of mosquito adaptive mutations, while viruses with double SLs show robustness during host switching.

The fitness advantage of viruses with SL deletions for replication in mosquito cells and the redundant function of the two SLs for replication in human cells (Fig. 5A) do not explain why the virus maintains two nearly identical RNA structures at the 3’UTR, a feature conserved in mosquito borne flaviviruses (see below). Therefore, we explored possible benefits of SL duplication during host switching. To emulate host switching events, viruses containing different modifications in the two SLs were tested for their ability to infect and replicate in different host cells. The first pair of viruses tested contained both SLI and SLII or only SLII, and in both constructs the SLII structure resembled that in mammalian-adapted variants. The second pair of viruses was identical to those just described, except that both contained a specific destabilizing mutation in SLII that mimicked a mosquito-adapted variant. Replication of these four viruses was evaluated in mammalian and mosquito cells. The mosquito-adaptive mutation in the virus with a single SL showed a drastic 48-fold reduction in viral replication in mammalian cells, as compared with its mammalian-adapted counterpart (Fig. 6A, right panel). In contrast, a virus with the same mutation but containing two SLs (Double SL) exhibited only 4-fold reduction in viral replication (Fig. 6A, left panel). This observation indicates that SL duplication allows the virus to replicate efficiently despite the presence of deleterious mutations associated with adaptation to mosquito cells. Interestingly, viruses adapted to mammalian cells carrying one or both SLs showed only a 3-fold difference in viral replication (Fig. 6A, compare mammalian-adapted viruses left and right panels), indicating that SL duplication is not as important in a virus that replicates in a single host, but becomes critical when the virus has to shuttle between two hosts. In other words, the fitness cost of mosquito-associated mutations in DEN-SLII for replication in mammalian cells is greatly alleviated by the presence of a second SL.

When the same experiment was repeated in mosquito cells, viruses containing mosquito-adaptive mutations in SLII exhibited superior levels of replication, while the difference between corresponding pairs with single or double SLs was negligible (Fig. 6B). These results suggest that viral replication in mosquito cells does not benefit significantly from SL duplication.

We conclude that maintaining two SLs gives the virus the ability to retain high fitness in both hosts even in the face of mosquito-adaptive mutations. The duplicated structure relaxes the sequence constraints on one of the SLs, allowing accommodation of mutations that are beneficial in only one environment (Fig. 6C).

### Duplication of SL structures is common in mosquito-borne flaviviruses

**Figure 7:**
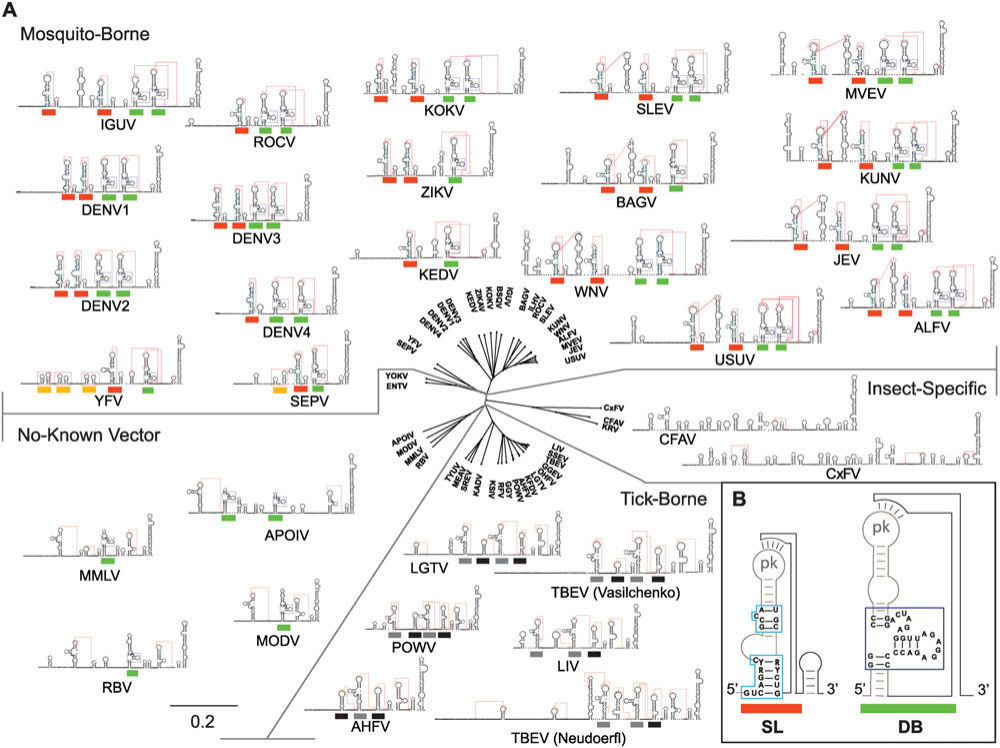
Models of flavivirus 3’UTR secondary RNA structures. **(A)** Comparative analysis of predicted RNA structures of members of the Flavivirus genus. Mosquito-borne, tick-borne, no-know vector and insect-specific flaviviruses are shown. Pseudoknots are indicated with red lines. Common SLs and DB structures are labeled with red and green lines, respectively. Specific yellow fever repeats are indicated with yellow lines and repeats in tick-borne flaviviruses with black and grey lines. See S1 Table for information on sources of sequences used for this analysis. The distance tree was drawn using neighbor joining method and jukes-cantor substitution model. **(B)** Conserved sequence of SL and DB structures common to all MBFVs are shown.

The relevance of SL duplication in DENV during host switch motivated us to examine the phylogenetic origin of RNA duplications in flavivirus 3’UTRs. First, we modeled the 3’UTR secondary structure of all available genomes of mosquito-borne, tick-borne, no-known vector and insect-specific flaviviruses. About 40 different flavivirus 3’UTR structures were predicted using RNAalifold, RNAaliduplex, and R software functions. A summary of the results from this comprehensive analysis is depicted in Fig. 7. From this study, conserved structural blocks of SLs and DBs were identified in all mosquito-borne flaviviruses (MBFV) (shown by red and green bars in Fig. 7A and B). These structures were absent in the 3’UTRs of insect-specific flaviviruses, from *Aedes* or *Culex* mosquitoes (Fig. 7A). In the case of flaviviruses with no-known vectors, no SL structures and single copies of DB elements were observed in most cases (Fig. 7). The 3’UTR of YFV contains single copies of conserved SL and DB structures. However, it contains duplications of structures specific to its group, described as YFV repeats (Fig. 7A, yellow bars). For tick-borne flaviviruses, duplications of RNA structures, different from SLs and DBs, were observed (Fig. 7A, black and grey bars), in agreement with previous reports [20]. This analysis indicates that RNA structure duplication is a common feature of flaviviruses that alternate between vertebrate and arthropod hosts. Importantly, the identified host-adaptable SL structure is both conserved and duplicated in most MBFVs.

## Discussion

Here, we demonstrate that the composition of the DENV population changes in different hosts. Adaptation studies in mosquito and mammalian cells revealed a process of host-specialization of a viral RNA structure. This RNA specialization has a significant fitness cost in the non-specialized host, posing a constraint during host switching. Interestingly, RNA structure duplication was found to reduce this fitness cost conferring higher levels of evolvability and capacity to accommodate mutations. We propose a new model of DENV 3’UTR evolution, where duplication of an RNA structure confers robustness by redundancy, allowing for rapid host-specific adaptation without significantly reducing viral fitness during host switch.

The high plasticity of the DENV 3’UTR observed during host adaptation was not a consequence of random drift or neutral mutations, but rather due to a selection process that led to significant changes in viral fitness in each host. Cycles of disruptions and reconstitutions of a specific SL RNA structure after host switching were observed. Under our experimental conditions, this process was facilitated by the presence of low abundance variants in the population. For instance, variants with an intact SL present at a frequency of about 2% increased to 89% of the population after switching from mosquito to mammalian cells (Fig. 2C). However, in nature, we speculate that bottlenecks would lead to different pathways of DEN-SLII reconstitutions in human cells. In support of this idea, natural DENV isolates showed extensive co-variations within DEN-SLII, indicating a strong evolutionary force to conserve the RNA structure regardless of the nucleotide sequence. Although DEN-SLI and DEN-SLII appear to have a common origin, they are under different selective pressures in nature. Sequence alignments and conservation studies indicate that DEN-SLI has low co-variations in comparison with DEN-SLII. It is important to mention that the nucleotides in the side loops of both SLs are highly variable because they appear to be under relaxed selection. In this regard, viral fitness was not affected by mutations or even deletions of these elements in both SLs (Fig. 5C).

Selection of specific RNA structures in different hosts was associated with DENV fitness gains (Figs. 2 and 5); however, the molecular cause of this selection is still unclear. In this regard, new functions of flavivirus 3’UTRs could be associated with host-specific activities. For instance the RNA structures were recently linked to: i) generation of subgenomic flavivirus RNAs (sfRNAs) [17], ii) production of viral-derived small RNAs [21, 22], and iii) interaction with host proteins, including those with RNA remodeling activity or linked to antiviral responses [23, 24]. sfRNAs are products of incomplete degradation of the viral genome by the cellular XRN1, which stalls at secondary RNA structures at the beginning of the 3’UTR [17]. In a recent report, a SL structure of the Murray Valley encephalitis virus 3’UTR, similar to SLI and SLII described for DENV, was crystalized and it was shown that its highly ordered RNA structure was responsible for sfRNA production [25]. Here, we found that replication of DENV in mosquito cells selects viral variants with RNA structures and PK disruptions that could modulate the XRN1 activity. Production of sfRNAs has been also shown to inhibit IFN-α/β activities in human cells and RNAi pathways in mosquito cells [26, 27]. Elucidating whether sfRNA production in mosquito and mammalian cells is the driving force for viral 3’UTR evolution in different hosts requires further investigation. Nevertheless, the selection of specific mutations in viral 3’UTR structures and the ability of these RNA elements to differentially modulate viral fitness in a host-specific manner represent a new mechanism of host adaptation of an RNA virus.

Evidence for an association of mutations in the variable region of DENV 3’UTRs with host-specific requirements was previously noticed using different viral serotypes [28, 29, 30]. Furthermore, changes in the DENV genome after sequential passages of DENV in mosquito and mammalian cells have been previously investigated. Although in this analysis consensus sequences of viral populations were used, positive selection of mutations in the 3’UTRs was observed [31]. Using our models of DENV 3’UTR structures those mutations were also mapped to the DEN-SLII, in agreement with our deep sequencing studies. Interestingly, the entire genome of a DENV-3 isolated from a pool of naturally infected *Ae. aegypti* sequenced during entomological surveillance in Brazil revealed deletions and insertions in the variable region of the viral 3’UTR [32]. In certain isolates, specific substitutions at positions U10391 and A10383 were found to disrupt DEN-SLII structure. These studies using different DENV serotypes are in agreement with the idea that the viral 3’UTR is under strong host-specific selective pressure. It is likely that 3’UTR sequence and structure duplications are also relevant for host adaptation of different arboviruses. In this regard, an interesting recent report investigated Chikungunya virus (CHIKV) 3’UTR evolution in different hosts [33]. In this case, duplication of direct repeats (DR) in the CHIKV 3’UTR were found to be beneficial for viral replication in mosquito cells, opposite to what we observed for DENV, in which 3’UTR deletions were positively selected in mosquito cells and mosquitoes. Based on these observations, there is a common theme that associates RNA structure duplications to host adaptation in arthropod-borne viruses, but likely these processes utilize different viral-specific mechanisms.

An association of direct repeats and RNA structure duplications with adaptation to multiples vertebrate and invertebrate hosts was previously proposed using a pan-flavivirus alignment [12]. In agreement with these observations, our RNA structure comparisons of flavivirus 3’UTRs revealed the conservation of SLs in most MBFVs (Fig. 7). MBFV include seven groups: YFV group, JEV group, DENV group, Aroa virus group, Kokobera virus group, Ntaya virus group, and Spondweni virus group [34]. Members of the JEV group, including WNV, SLEV, JEV and MVEV; members of the DENV group, including serotypes 1, 2 and 3, and most of the members of the Aroa, Kokobera, Ntaya, and Spondweni groups contained a duplication of the host-adaptable SL element. In addition to DENV4, ROCV also contains a single SL and a duplicated DB, while KEDV bears a single SL and a single DB structure (Fig. 7). The 3’UTR variable region of members of the JEV group is more complex than that in the DENV group, containing three or four structures previously described in WNV as SLI, SLII, SLIII and SLIV [17]. However, only SLII and SLIV contain the common structural elements of SLs described here for DENV and conserved among different flaviviruses (Fig. 7A and B). Although RNA structure duplications are widely conserved in MBFV genomes, no experimental evidence for function of this duplication has been previously described. Here, we provide the first functional link between RNA structure duplication and host adaptation. We found that variants with two SLs display high fitness in mammalian cells even in the presence of mosquito-adaptive mutations, while these mutations in a virus with a single SL drastically reduced viral replication in the same system. We propose that SL structure duplication increases viral robustness for a successful viral transition from mosquito to human. DENV also circulates and evolves through a sylvatic cycle between mosquitoes and non-human primates. In this regard, sequences of viral isolates from mosquitoes captured at sylvatic habitats showed selection of mutations disrupting the SL structure (Fig. 4C), suggesting that similar RNA host adaptations occur in sylvatic cycles. We conclude that duplication of an adaptable RNA element that is under conflicting host-selective pressures represents a new mechanism of sustaining high viral fitness in a virus that must cycle between hosts. These studies provide a novel paradigm for better understanding both viral RNA evolution and host adaptation.

## Materials and Methods

### Viruses, transfections, infections and fitness measurements

DENV infections were performed using different cell lines as indicated in each case. Baby hamster kidney cells (BHK-21, ATCC, CCL-10) and human lung cells (A549, ATCC, CCL-185) were grown at 37°C in MEM alpha and D-MEM media, respectively. Mosquito C6/36HT and U4.4 cells were grown at 33°C and 28°C, respectively, in Leibovitz L-15 medium.

Viral stocks were obtained by transfection of 5 μg of in vitro transcribed viral RNA using lipofectamine. Supernatants were harvested at different times post-transfection and viruses were quantified by plaque assays as previously described [28]. For one step growth curves, BHK-21 or C6/36 cells in six-well plates were infected at moi of 0.1 for DENV-WT or DENV-Mut1, and recovered viruses were quantified by plaque assays in BHK cell. Immunofluorescence assay (IFA) using specific DENV antibodies against the envelope protein were used as previously described [28].

### Construction of recombinant DENVs

Mutations were introduced into the full-length cDNA of DENV 2 pD2/IC AflII, replacing the Afl*II*-Xba*I* fragment of the WT plasmid with a fragment derived from overlapping PCRs containing the desired mutation as previously described [28]. For reporter virus studies, the mutations were introduced in the context of the mDV-R replicon virus [19].

### Mosquito rearing and infections

*Aedes albopictus Lake Charles* strain laboratory mosquitoes originating from Louisiana were reared from eggs and maintained as adults at 28°C, and 80% relative humidity with a photocycle of 12 h light:12 h dark, and given water and sugar until infection. Adult female mosquitoes 5 days post-emergence were intrathoracically (IT) inoculated with approximately 70 nl cell culture medium containing a 9:1 ratio of WT:M132 DENV stock with a total titer of 200 PFU, using a Nanojet II (Drummond Scientific Company). To ensure productive infection with defined amounts of viruses in the mix for competition experiments, mosquitoes were infected by IT injection rather than oral blood-meal. Mosquitoes were maintained for 10 days and used for RNA extractions. Each infection experiment was carried out in triplicate.

### Experimental host adaptation and sequencing

In vitro transcribed DENV RNA was transfected into the cell lines described above. Two independent RNA transfections were performed for each adaptation experiment. Viruses were harvested at 3 days (P1) and quantified by plaque assay. Successive infections in the same cell line or in the alternate host cell were performed at moi of 1. Two P10 and two P20 populations that were passage independently were used for viral RNA purification. Viral RNAs were Trizol-extracted from culture supernatants and used for RT-PCR reactions in duplicates with tagged primers specifically designed to amplify the last 600 nucleotides of both genome ends. The in vitro transcribed viral RNA was also sequenced as reference (IP). Libraries were sequenced using a 454 Genome Sequencer FLX Titanium XLR70 System and raw data was analyzed as describe below. The two P10 populations obtained from mosquito cells, the two P10 from mammalian cells and one P20 from both were used for cloning and sequencing.

### Sequencing data analysis

Data exploration and processing were performed using R environment (R Development Core Team, 2013) with base packages, Biostrings packages and developed R functions. Reads covering 200 nucleotides of the 3’ and 5’UTR were aligned to the reference sequence. Technology generated insertion were manually filtered by comparison with the input RNA control as previously described [35]. To define selected viral variants, the sequence vector space approach was applied [36]. For each alignment a comparative matrix was constructed and dimensional reduction of matrices were made. Ten principal components were used to determine the clusters of sequences using “Density-based clustering”. Finally, the prototype sequence of each cluster corresponding to each variant was the consensus sequence.

### RNA structure determination and predictions

To study the secondary and tertiary RNA structures of the whole 3’UTR, selective 2’-hydroxyl acylation analyzed by primer extension (SHAPE) was used as previously reported [37, 38], employing as template fragments of 600 nucleotides or the complete 11 kb viral RNA genome.

For conservation analysis of RNA structures, DENV isolates representative of different genotypes and isolates corresponding to mosquito-borne, tick-borne, insect-specific and mammalian-only flaviviruses were used. Detailed information of GenBank ID references of sequences used throughout this work is shown in S1 Table. Each group of sequences was aligned using Clustal and manually corrected. The RNAz and RNAalifold softwares were used to detect thermodynamically stable and evolutionarily conserved RNA secondary structures [39, 40]. To characterize possible tertiary interactions, complementary base pairs and co-variations, we used Biostring functions and the RNAaliduplex software [40] together with predicted secondary structure models. In the case of viruses with a single GenBank report, sequences were added to alignments of related flaviviruses and comparative secondary structure predictions were performed taking into account conserved features and previous predictions.

## Supporting Information

S1 Fig Nucleotide sequence of DENV 3’UTRs of different population. Alignment of the nucleotide sequence corresponding to the first 160 nucleotides of the 3’UTR from deep sequencing experiments is showed. Information of variant frequency and amount of nucleotide changes is indicated on the right of each sequence.
(EPS)

S2 Fig Sequence variations at the DENV UTRs during experimental host adaptation. (A) Deep sequencing of viral populations after 10 passages (P10) in mosquito and mammalian cells. The data corresponds to 3’UTR analysis of an independent adaptation experiment as the one shown in Fig. 1A. (B) Alignment of the nucleotide sequence corresponding to the first 160 nucleotides of the 3’UTR of populations shown in A. (C) Sequence variations at the DENV 5’UTR during experimental host adaptation.
(EPS)

S3 Fig DENV host adaptation experiments. (A) Alignment of the nucleotide sequence corresponding to the first 160 nucleotides of the 3’UTR of viral populations adapted to mosquito C6/36 (experiments I, II, and III) and U4.4 cells. Information of variant frequency and amount of nucleotide changes is indicated on the right of each sequence. (B) Schematic sequence alignment from conventional sequencing of cloned amplicons corresponding to the complete 3’UTR of viral populations adapted to BHK cells. The input sequence is presented at the top. Three experiments are shown. Detected changes are indicated in red and a conservation plot is presented at the bottom.
(EPS)

S4 Fig Sequence variations of DENV 3’UTRs after host switch. (A) Detailed nucleotide sequences of viral populations, before (top) and after (bottom) switch to mammalians cells, is presented. (B) Nucleotide sequences of mammalian cell adapted virus, before (top) and after (bottom) switch to mosquito cells, is showed. Information of variant frequency and amount of changes is indicated on the right of each sequence.
(EPS)

S5 Fig Properties of the variable region of DENV4. (A) Representation of the unique SL RNA structure of DENV4 from natural human isolates corresponding to different genotypes. Sequence alignment plot and secondary RNA structure model are shown. (B) Schematic representation of reporter DENV containing the luciferase gene carrying different 3’UTRs as indicated. (C) RNAs of reporter DENVs corresponding to the parental DENV2, a chimeric virus containing the variable region of DENV4 (ChDENV2) and a ChDENV2 containing a mutations at the top loop disrupting the PK (Mut-ChDENV2) were transfected into C6/36 and BHK cells. Normalized luciferase levels are shown using a logarithmic scale at 28 and 48h post transfection. The luciferase values are the mean +/- SD, n = 4.
(EPS)

S1 Table Flavivirus nucleotide sequences used in this study. (XLS)
